# Supplementary material for: Time-series transcriptome analysis identified differentially expressed genes in broiler chicken infected with mixed Eimeria species
Source: Front Genet. 2022 Aug 8;13:886781. doi: 10.3389/fgene.2022.886781 (PMC9393255; doi:10.3389/fgene.2022.886781)
Supplement: Supplementary file 2 [file DataSheet1.ZIP › 4dpi_KEGG.Gsea.1625100298584/KEGG_TYROSINE_METABOLISM.html]

Details for gene set KEGG\_TYROSINE\_METABOLISM[GSEA]

|  || Dataset | TMM\_4dpi\_gct\_format\_4dpi\_gct\_format.Class\_4dpi.cls #PC\_versus\_NC.Class\_4dpi.cls #PC\_versus\_NC\_repos |
| Phenotype | Class\_4dpi.cls#PC\_versus\_NC\_repos |
| Upregulated in class | 0 |
| GeneSet | KEGG\_TYROSINE\_METABOLISM |
| Enrichment Score (ES) | -0.43329754 |
| Normalized Enrichment Score (NES) | -1.3248397 |
| Nominal p-value | 0.12337371 |
| FDR q-value | 0.39167443 |
| FWER p-Value | 0.9998 |
Table: GSEA Results Summary

  

Fig 1: Enrichment plot: KEGG\_TYROSINE\_METABOLISM      
 Profile of the Running ES Score & Positions of GeneSet Members on the Rank Ordered List

  

| SYMBOL | TITLE | RANK IN GENE LIST | RANK METRIC SCORE | RUNNING ES | CORE ENRICHMENT || 1 | HEMK1 | na | 442 | 1.022 | 0.0681 | No |
| 2 | ALDH1A3 | na | 581 | 0.895 | 0.1484 | No |
| 3 | DDC | na | 1072 | 0.648 | 0.1741 | No |
| 4 | MAOB | na | 3877 | 0.180 | -0.0414 | No |
| 5 | GOT1 | na | 3928 | 0.174 | -0.0276 | No |
| 6 | MAOA | na | 5750 | 0.013 | -0.1782 | No |
| 7 | GOT2 | na | 5770 | 0.012 | -0.1786 | No |
| 8 | AOC3 | na | 5873 | 0.004 | -0.1867 | No |
| 9 | METTL2B | na | 6336 | -0.033 | -0.2219 | No |
| 10 | TRMT11 | na | 7216 | -0.110 | -0.2839 | No |
| 11 | MIF | na | 7520 | -0.136 | -0.2952 | No |
| 12 | GSTZ1 | na | 7777 | -0.159 | -0.3002 | No |
| 13 | IL4I1 | na | 8390 | -0.217 | -0.3289 | No |
| 14 | ADH6 | na | 9642 | -0.367 | -0.3956 | Yes |
| 15 | FAH | na | 9655 | -0.368 | -0.3588 | Yes |
| 16 | COMT | na | 9712 | -0.376 | -0.3249 | Yes |
| 17 | LCMT2 | na | 9771 | -0.382 | -0.2905 | Yes |
| 18 | ALDH3B2 | na | 10162 | -0.439 | -0.2780 | Yes |
| 19 | LCMT1 | na | 10251 | -0.453 | -0.2389 | Yes |
| 20 | METTL6 | na | 10938 | -0.591 | -0.2355 | Yes |
| 21 | AOX1 | na | 11728 | -0.999 | -0.1988 | Yes |
| 22 | ADH4 | na | 11768 | -1.035 | -0.0958 | Yes |
| 23 | ADH1C | na | 11810 | -1.129 | 0.0167 | Yes |
Table: GSEA details [plain text format]

  

Fig 2: KEGG\_TYROSINE\_METABOLISM      
 Blue-Pink O' Gram in the Space of the Analyzed GeneSet

  

Fig 3: KEGG\_TYROSINE\_METABOLISM: Random ES distribution      
 Gene set null distribution of ES for **KEGG\_TYROSINE\_METABOLISM**
